# The Relationship Between Physical Activity and Quality of Life During the Confinement Induced by COVID-19 Outbreak: A Pilot Study in Tunisia

**DOI:** 10.3389/fpsyg.2020.01882

**Published:** 2020-08-07

**Authors:** Maamer Slimani, Armin Paravlic, Faten Mbarek, Nicola L. Bragazzi, David Tod

**Affiliations:** ^1^ Department of Health Sciences (DISSAL), Postgraduate School of Public Health, University of Genoa, Genoa, Italy; ^2^ Science and Research Centre, Institute for Kinesiology Research, University of Primorska, Koper, Slovenia; ^3^ Department of Sociology, Higher Institute of Applied Studies in Humanities of Gafsa, Gafsa, Tunisia; ^4^ Laboratory for Industrial and Applied Mathematics (LIAM), Department of Mathematics and Statistics, York University, Toronto, ON, Canada; ^5^ School of Sport and Exercise Sciences, Liverpool John Moores University, Liverpool, United Kingdom

**Keywords:** coronavirus, COVID-19, prevention, psychosocial, confinement

## Abstract

The present study aimed to determine the relationship between physical activity (PA) and quality of life (QoL) during the confinement caused by the COVID-19 outbreak. A total of 216 participants (men: *n* = 112, women: *n* = 114) were included in the present study. They were divided into three groups [i.e., inactive group (IG): less than 600 metabolic equivalent of tasks (METs), *n* = 131; minimally active group (MAG): from 600 to 2,999 METs, *n* = 49; and health-enhancing PA group (HEPAG): 3,000 + METs, *n* = 36] based on their habitual PA level in the period of confinement. WHO Quality of Life Instrument-Short Form (WHOQOL-BREF) and International Physical Activity Questionnaire-BREF (IPAQ-BREF) questionnaires were used to assess QoL and PA intensities. The main findings of the present study showed that MAG and HEPAG have better total PA, physical, psychological, social, and environmental QoL domains scores than IG (all, *p* < 0.01). Small to large correlations (*r* ranging from 0.14 to 0.72) were also observed between total PA, total walking activity, total moderate-intensity PA, total vigorous-intensity PA, and QoL domains (all, *p* < 0.01). PA with light-, moderate-, and vigorous-intensities can be well recommended to decrease the negative psychosocial effect of confinement. However, longitudinal studies are needed to draw causal inferences and underpin more robust and evidence-based and informed recommendations.

## Introduction

“Severe Acute Respiratory Syndrome coronavirus type 2 (SARS-CoV-2),” responsible for an infection termed the coronavirus disease (COVID-19), is a newly discovered pathogen in humans. It was first discovered on November 17, 2019 in the city of Wuhan, mainland China, and then spread throughout the world. On March 11, 2020, COVID-19 was declared a pandemic by the World Health Organization (WHO). As of 24 March 2020, the number of confirmed cases was 372,755 cases and 16,231 deaths, which increased to 2,160,207 cases and 146,088 deaths as of 18 April 2020 (WHO Situation reports; Zhu et al., 2020). In this context, the WHO recommended the adoption of various protective, behavioral, and non-pharmacological measures (such as avoiding physical contact, handshakes, hugs and kisses; banning social gatherings and major events; closing universities and schools; and implementing self-isolation, social/physical distancing, confinement, and quarantine) to curb the spread of the virus as well as preventive approaches [including practicing physical activity (PA), sleeping well, etc.] to keep oneself healthy during the ongoing outbreak.

Governments of all countries ordered their people to self-isolate (stay at home). However, long-term isolation or home-confinement may have negative effects on psychosocial and mental health, especially causing stress, negative emotions, and impaired cognition (Hawkley and Capitanio, 2015). If prolonged, they may suppress immune system and physiological functions (Kiecolt-Glaser et al., 2002), which may increase the risk of exposure to SARS-CoV-2 and the likelihood of contracting the infection. For instance, the WHO suggested that people stay physically active at home in order to optimize their health status, decrease the negative psychosocial consequences of confinement, and maintain their immune system function.

Many studies have reported positive effects of PA on psychosocial status, such as quality of life (QoL), outside of confinement (Anokye et al., 2012; Vagetti et al., 2014; Krzepota et al., 2018; Ho et al., 2019). However, no study has so far investigated the relationship between PA and QoL during a period of confinement, such as the quarantine caused by COVID-19. Therefore, we aimed to explore the relationship between PA and QoL in the general population of Tunisia during the first 4 weeks of the confinement implemented by the government to curtail the COVID-19 pandemic. This may help the community members to improve their QoL in the face of a future pandemic, such as the COVID-19 outbreak or potential secondary waves/relapses, should the virus not be completely eradicated and suppressed by means of pharmacological interventions (drugs and vaccines).

## Materials and Methods

### Participants

A non-probabilistic sampling approach was utilized. Subjects were considered eligible to take part into the study if meeting the following inclusion criteria: (i) age in the range 18–30 years; (ii) not using alcohol, drugs, or other substances; and (iii) no co-morbidities and/or orthopedic limitations that could interfere with the perceived QoL or level of PA.

A link to the online questionnaires was sent *via* mail to 242 potential participants, of which 216 returned valid questionnaires (participation rate of 89.3%). Thus, 216 participants were included in the analysis. The link contained a brief explanation of the questionnaires and instructions on how to complete them. It was sent 4 weeks after the implementation of the quarantine by the Tunisian government. All measures were collected on the same day, to avoid any bias in the study, considering the constantly evolving situation of the pandemic.

Most participants in the current survey were men (*n* = 112, 51.9%), and the mean age at the time of the study was 27.9 years (*SD* = 8.1). Participants were divided into three groups [i.e., inactive group (IG): less than 600 metabolic equivalent of tasks (METs), *n* = 131; minimally active group (MAG): from 600 to 2,999 METs, *n* = 49; and health-enhancing PA group (HEPAG): 3,000 + METs, *n* = 36] based on their habitual PA level in the period of quarantine, as recommended by previous research (Lee et al., 2011).

We excluded participants who were not compliant to government guidelines at home during the COVID-19 epidemic. Participants completed the online PA and QoL questionnaires. They were thoroughly advised of the aims of the study. All participants signed a free and informed consent form. Local institutional ethical approval was provided for this study, which was conducted in accordance with the 1964 Declaration of Helsinki and its subsequent amendments.

### Measures

#### Physical Activity

PA was assessed using the International Physical Activity Questionnaire-BREF (IPAQ-BREF; Lee et al., 2011). This questionnaire comprised of seven questions which assessed the frequency and duration of vigorous intensity, moderate intensity, and walking PA for at least 10 min during the past week. Participants were asked to respond to one of the frequency options: from 1 to 7 days. Duration options included the number of minutes exercised, never do, or do not know. Participants who had not undertaken any PA in the previous 7 days responded only to the question “During the last 7 days, how much time did you spend sitting on a week day?”

The IPAQ assessed total PA and total sedentary time, whereas the intensity of activity was converted to MET units (MET^-h^·week^−1^), as recommended by previous study (Ainsworth et al., 2000).

#### Quality of Life

QoL was measured using the self-administered WHO Quality of Life Instrument-Short Form (WHOQOL-BREF; Skevington et al., 2004). It comprised 26 items including domains and facets (or sub-domains). The first two items assessed the “overall rating of QoL (OQOL)” and subjective satisfaction with health. The other 24 items measured four domains, namely, physical health (seven items), psychological health (six items), social relations (three items), and environment (eight items). The participants marked a response using a 5-point Likert scale [ranging from 1 (very dissatisfied/very poor) to 5 (very satisfied/very good)]. The domain scores of the WHOQOL-Bref were computed according to the guideline of the WHO (WHO, 1998).

### Statistical Analysis

Statistical analyses were performed by means of the commercial software “Statistical Package for the Social Sciences” (SPSS for Windows, version 20.0, SPSS Inc., Chicago, Illinois, USA). Descriptive statistics were calculated for all experimental data. Kolmogorov-Smirnov test was used to assess if data were normally distributed. Differences in PA level and QoL domains were determined by using Mann-Whitney U tests. To assess a difference between different categories of participants based on their PA level, Kruskal-Wallis tests were used. In the case of significant differences, *post hoc* comparisons with Bonferroni corrections were applied using the Mann-Whitney U test. Furthermore, bivariate correlations were computed by using Spearman’s Rho to examine the relationship between PA level and QoL domains.

## Results

An overview of the participants’ METs and QoL domain scores is shown in Table 1. Most of the time was spent with participants being sedentary, followed by light-, moderate-, and high-intensity activities, regardless of the group. In general, all dependent variables differed among the groups (Kruskal-Wallis test, *p* < 0.001; Table 1), except being sedentary. *Post hoc* analyses revealed that the differences between groups were significant in all comparisons and by the pattern, where the IG accumulated less light-, moderate-, and high-intensity activities when compared to HEPAG (Table 1). When compared to the MAG, the IG did not differ in total time spent at light activities only.

**Table 1:** Differences in the levels of physical actvity (PA) as assessed by means of the International Physical Activity Questionnaire (IPAQ) and perceived quality of life (QoL) between the different groups recruited in the present study.

Variables	IG *n* = 131	MA group *n* = 49	HEPA *n* = 36	Kruskal-Wallis test	IG vs. MA Z (*p* value)	IG vs. HEPA Z (*p* value)	MA vs. HEPA Z (*p* value)
Age	28.2 ± 8.4	24.8 ± 4.9	27.9 ± 9.7	0.097	-	-	-
Total PA (MET)	39.2 ± 122.9	1630.3 ± 680.7	6074.4 ± 2993.4	<0.001	−12.053 (<0.001)	−11.255 (<0.001)	−7.845 (<0.001)
WHOQoL physical domain score	40.6 ± 10.9	47.5 ± 16.1	49.3 ± 11.9	<0.001	−3.448 (0.001)	−4.484 (<0.001)	−0.063 (0.950)
WHOQoL psychological domain score	41.6 ± 12.3	57.4 ± 11.3	60.6 ± 10.1	<0.001	−6.578 (<0.001)	−6.858 (<0.001)	−1.102 (0.270)
WHOQoL social domain score	43.5 ± 11.3	62.4 ± 21.6	64.6 ± 13.1	<0.001	−5.882 (<0.001)	−7.270 (<0.001)	−0.816 (0.415)
WHOQoL environmental domain score	41.0 ± 13.5	53.4 ± 16.2	55.6 ± 15.6	<0.001	−4.894 (<0.001)	−4.816 (<0.001)	−0.504 (0.614)
WHOQoL total score	166.7 ± 38.2	221.6 ± 49.9	230.1 ± 34.9	<0.001	−6.057 (<0.001)	−6.966 (<0.001)	−0.441 (0.659)

Data are presented as mean ± SD. HEPA, health enhancing physical activity; IG, inactive group; MA, minimally active; MET, metabolic equivalent of task; PA, physical activity; WHOQOL, World Health Organization Quality of Life.

Regarding QoL, the IG reported lower scores compared to the MAG and HEPAG for all four domains, while the MAG and HEPAG did not differ (*p* = 0.950). The same pattern was observed for QoL total score, where the IG reported lower scores compared to the MAG and HEPAG, while the MAG and HEPAG did not differ (*p* = 0.659; Table 1).

Correlation analysis showed that total PA (MET) had a small to moderate relationship with QoL domains (Table 2). More in-detail, small to moderate correlations (*r* ranging from 0.14 to 0.42) between all PA intensities and QoL domains were observed (Table 2).

**Table 2:** Coefficients showing the strength of the association between PA and the WHOQOL domain scores for the sample recruited (*n* = 216).

Variables	Total walking activity (MET)	Total moderate-intensity activity (MET)	Total vigorous-intensity activity (MET)	Total sedentary time	Total PA (MET)
WHOQoL physical domain score	0.196**	0.170*	0.187^**^	0.006	0.232^**^
WHOQoL psychological domain score	0.401^**^	0.354^**^	0.264^**^	0.201^**^	0.418^**^
WHOQoL social domain score	0.422^**^	0.312^**^	0.242^**^	0.025	0.407^**^
WHOQoL environmental domain score	0.265^**^	0.249^**^	0.141^*^	0.042	0.263^**^

MET, metabolic equivalent of task; PA, physical activity; WHOQOL, World Health Organization Quality of Life. ^*^ *p* < 0.05, ^**^ *p* < 0.001.

## Discussion

The present study aimed to determine the relationship between PA and QoL during the confinement caused by the COVID-19 outbreak. The main findings of the present study showed that MAG and HEPAG groups have better total PA, physical, psychological, social, and environmental domains scores than the IG. Small to large correlations were also observed between total PA, total walking activity, total moderate-intensity PA, total vigorous-intensity PA, and the QoL domains.

The relationship between active living and QoL is well studied in patients with different diseases and also in healthy participants (Ellingson and Conn, 2000; Brown et al., 2003; Anokye et al., 2012; Ha et al., 2014; Krzepota et al., 2018; Ho et al., 2019). However, the current study showed that total PA (MET) was correlated with all QoL domains during a period of government directed confinement and a limiting of personal freedom. Specifically, large correlations were observed between total PA and psychological and social QoL domains. Accordingly, some studies have also reported a positive correlation between PA and QoL domains (Mourady et al., 2017). For instance, some authors have reported positive correlations between total PA and physical and mental domains (Stewart et al., 2003; Wendel-Vos et al., 2004; Fox et al., 2007; Shibata et al., 2007), while others have shown positive correlations between PA and some QoL domains, namely general QoL; functional capacity; mental health; autonomy, past, present, and future activities; death and dying; intimacy; vitality; and psychological domain (Vagetti et al., 2014). Mourady et al. (2017) reported that total PA was significantly correlated with physical and psychological health, general QoL, social relationships, and the environmental domains in healthy participants. On the contrary, in one study, sedentary PA was significantly associated with the social relationship domains (Mourady et al., 2017). The last finding is not in agreement with our study result regarding the correlation between sedentary and QoL domains (e.g., in our study total sedentary mostly had non-significant relationships with QoL domains). This contradiction may be explained by the differences in the study contexts. Participants in the present study practiced social distancing to avoid the spread of COVID-19. In addition, by staying at home, without practicing PA, they may feel insecure.

Regarding the dose-response relationship between PA and QoL domains, the current study reported small to moderate correlations between all PA intensities and QoL domains. More in-detail, moderate correlations were observed between low- and moderate-intensities and psychological and social domains. Previous studies have reported positive relationships between moderate to vigorous PA and several SF-36 domains in participants attending a behavior change service within primary care, with these domains including: (a) general health and vitality, (b) physical functioning, and (c) the role of physical activity (Blom et al., 2019). Light PA was also associated with the aforementioned domains and the emotional sphere (Blom et al., 2019). This is not surprising, since a lot of studies showed the beneficial effect of light, moderate, and vigorous physical activities on all QoL domains (Gillison et al., 2009; Gill et al., 2013).

Regarding the correlation between PA and QoL domains, in our study, total PA, total walking activity, total moderate-intensity PA, and total vigorous-intensity PA were positively correlated with all QoL domains in both males and females. These findings are in agreement in part with Nakamura et al. (2014), who reported that leisure-time PA, moderate-intensity PA, and vigorous-intensity PA were associated with physical health domains. Moderate intensity and total activity leisure-time PA were also correlated with mental health in men (Nakamura et al., 2014). Van den Berg et al. (2008) reported that only vigorous-intensity PA was associated with the physical and mental health domains in workers. However, our study showed large correlations between psychological and social domains and all intensities of PA. In addition, a previous study reported that light-intensity of PA was positively correlated with psychological health and social relationship domains in healthy participants (Mourady et al., 2017). These studies and the current investigation support the latest guidelines issued by the WHO suggesting that people attain 150 min of moderate-intensity, 75 min of vigorous-intensity PA per week, or a combination of both to improve health, well-being, and QoL during the confinement.

Despite its novelty and methodological rigor, the present study is not without any limitations that should be properly acknowledged. The major limitation is given by the sampling approach, which calls up for caution when interpreting and generalizing the results. A further drawback is represented by the study design that being cross-sectional does not allow causal inferences to be drawn from the data. Given the preliminary nature of the present report, further research is needed to confirm our conclusions among different populations, using representative samples. In particular, longitudinal studies are needed to better understand the relationship between PA levels and perceived QoL during the confinement measures and after their lifting.

From the present findings, we can conclude that there is an association between PA levels and perceived QoL during the confinement period and the COVID-19 outbreak. If longitudinal studies replicate our data, PA with light-, moderate-, and vigorous intensities can be well recommended as an important method to improve QoL and to decrease/counteract the negative psychosocial effects of confinement. However, based on the above-mentioned limitations, along with the impact that disease-caused confinement has on people physically and psychologically, further research in the field is urgently warranted.

## Data Availability Statement

The datasets presented in this article are readily available from the corresponding author (maamer2011@hotmail.fr).

## Ethics Statement

The studies involving human participants were reviewed and approved by local institutional ethical approval provided for this study, which was conducted in accordance with the 1964 Declaration of Helsinki. Written informed consent to participate in this study was provided by the participants’ legal guardian/next of kin.

## Author Contributions

All authors: conceptualization and methodology. MS and AP: software. All authors: validation and formal analysis. MS: investigation and resources. MS and AP: data curation. All authors: writing, original draft preparation, review and editing, and visualization. DT: supervision. NB: funding acquisition. All authors contributed to the article and approved the submitted version.

### Conflict of Interest

The authors declare that the research was conducted in the absence of any commercial or financial relationships that could be construed as a potential conflict of interest.
